# Invasion Dynamics of A Termite, *Reticulitermes flavipes,* at Different Spatial Scales in France

**DOI:** 10.3390/insects10010030

**Published:** 2019-01-15

**Authors:** Elfie Perdereau, Guillaume Baudouin, Stéphanie Bankhead-Dronnet, Zoé Chevalier, Marie Zimmermann, Simon Dupont, Franck Dedeine, Anne-Geneviève Bagnères

**Affiliations:** 1 IRBI, UMR 7261 CNRS-Université de Tours. Avenue Monge, Parc Grandmont, Tours 37200, France; baudouin.guillaume1@gmail.com (G.B.); zchevalier@hotmail.fr (Z.C.); marie.zimmermann@univ-tours.fr (M.Z.); simon.dupont@univ-tours.fr (S.D.); franck.dedeine@univ-tours.fr (F.D.); 2 LBLGC, INRA, Université d’Orléans, USC 1328, Orléans 45067, France; stephanie.bankhead@univ-orleans.fr; 3 CEFE, CNRS, Univ Montpellier, Univ Paul Valéry Montpellier 3, EPHE, IRD, Montpellier 34000, France; ag.bagneres@cefe.cnrs.fr

**Keywords:** biological invasions, expansion front, social insects, breeding system, phylogeography, genetic structure

## Abstract

Termites are social insects that can also be major pests. A well-known problem species is the subterranean termite, *Reticulitermes flavipes*. It is invasive in France and is thought to have arrived from Louisiana during the 18th century. While the putative source of French populations has been identified, little is known about how the termite spread following its establishment. Here, we examined expansion patterns at different spatial scales in urban areas to clarify how *R. flavipes* spread in France. Based on our analyses of phylogeography and population genetics, results suggest a scenario of successive introductions into the Charente-Maritime region, on the Atlantic Coast. Two major expansion fronts formed: one that spread toward the northeast and the other toward the southeast. At the regional scale, different spatial and genetic distribution patterns were observed: there was heterogeneity in Île-de-France and aggregation in Centre-Val de Loire. At the local scale, we found that our three focal urban sites each formed a single large colony that contained several secondary reproductives. Our findings represent a second step in efforts to reconstruct termite’s invasion dynamics. They also highlight the role that may have been played by the French railway network in transporting termites over long distances.

## 1. Introduction

The invasion process can be divided into a series of stages during which there are barriers that must be overcome for a population to become invasive [1,2]. Understanding these intricacies is an important part of predicting and managing biological invasions [3]. During the first stage, the transport and introduction of individuals into a new geographical range has a decisive impact on the diversity and genetic structure of introduced populations [4,5,6,7,8]. During the second stage, two factors will determine whether a viable population can be established: the ability of these individuals to survive new environmental conditions and to attain an adequate effective population size to allow a sustainable population to be founded [1,2,7]. During the last stage, the invasive population will display demographic and spatial expansion, which is rooted in a strong capacity to reproduce and disperse [4,9]. The process may be accelerated by additional factors, such as human-mediated transport and mechanisms promoting long-distance dispersal [10,11,12,13]. Recently, Roques et al. [14] carried out a detailed analysis of six invasive species and demonstrated that long-distance translocation associated with anthropogenic activities play a major role in determining rates of spread. Consequently, the spread of an invasive species is a complex process with significant effects on population spatial distribution and genetic structure within the introduced range [15].

*Reticulitermes flavipes* is an invasive subterranean termite (Rhinotermitidae) that lives in forests and urban areas, where it can cause significant damage to wooden structures of human origin [16]. It is native to the US, where it can be found from the eastern part of country (Massachusetts to Florida) to the Midwest and South (Nebraska and Texas). It has been unintentionally introduced into other countries in the Americas (i.e., the Bahamas, Canada, Chile, and Uruguay) and in Europe (i.e., Austria, France, Germany, Italy, and Canary Islands) [17,18]. A recent study of the termite’s genetics revealed that French populations (formerly known as *R. santonensis*) most likely came from an area in or around New Orleans, Louisiana [17]. It has been hypothesized that *R. flavipes* was accidentally introduced to France during the 18th century, when Louisiana was a French territory, arriving in shipments of agricultural products and/or timber [17,19]. *Reticulitermes flavipes* is now well established in France: its range extends over the western half of the country, from Paris to Marseille. It is common in urban areas but also occurs in certain pine forests along the Atlantic coast. Rochefort and La Rochelle were the first two cities in which termite damage to anthropogenic structures was reported—they are two major ports along the Atlantic Coast that played an important role in international trade during the period mentioned above [20,21]. *Reticulitermes flavipes* remains extremely widespread and abundant in urban and natural areas in and around these two cities. All of the evidence points to the following scenario: individuals of *R. flavipes* first arrived in one or several ports along the Atlantic Coast on ships coming from Louisiana. They reproduced and established themselves before spreading to other cities and expanding into local forests. If such is the case, the French populations of *R. flavipes* have been evolving independently for about 200 years [17].

In temperate habitats, termites of subterranean genera such as *Reticulitermes* display a high degree of invasiveness and frequently infest anthropogenic structures [22,23,24]. Subterranean termites have cryptic nesting habits and form complex colonies whose diffuse nests and multiple feeding sites are connected by underground tunnels. New colonies are typically founded by a pair of primary reproductives (i.e., winged adults, one queen, and one king) that mated after swarming. In the royal couple’s progeny, sterile individuals (i.e., workers, soldiers, larvae, and young nymphs) can differentiate into secondary reproductives [25], also known as neotenics (i.e., non-winged reproductives), which can interbreed and reproduce within their parental colonies [26]. This process of differentiation can occur either following the death of one or two primary reproductive(s) or during colony growth and expansion. Indeed, the presence of neotenics allows budding, which is an alternative mode of colony foundation. Furthermore, in some cases, different colonies can fuse into a single social unit that contains several unrelated reproductives and that covers a broad spatial area [27,28]. It is therefore clear that colony breeding structure directly influences colony growth and dispersal in *Reticulitermes* species, which then affect termite population dynamics.

Interestingly, the ability of colonies to produce neotenics seems to be highly variable among species and populations [16]. Research on *R. flavipes* populations has highlighted the relationship between invasion success and colony breeding structure [29]. Whereas native populations (i.e., in the US) are mainly composed of colonies headed by monogamous pairs of primary reproductives, the presumed source population (i.e., in New Orleans) and introduced populations (e.g., in France and Chile) tend to exhibit a unique colony breeding structure, in which there are hundreds of related neotenics, and where colonies show an unusual propensity to fuse. In France, this breeding structure is associated with the colonies’ spatial breadth, which commonly exceeds several hundred meters [28,29,30,31]. These two colony-level traits are thought to be preadaptations, allowing termite colonies to successfully invade new ranges, mainly because these traits allowed introduced colonies to reproduce and become established right after their introduction and then spread more rapidly via human-mediated dispersal [29]. Recently, researchers have developed temporal and spatial models integrating these traits that have been used to predict termite’s future spread [32]. More specifically, these studies aim to predict what will happen under conditions of climate warming in the Centre-Val de Loire region of France, where the termite’s presence was first recorded in the 1980s [32]. The results show that increasing temperatures should increase the amount of favorable habitat and, consequently, termites could continue to spread within this region and throughout France.

The objective of this study was to characterize the post-establishment dynamics of *R. flavipes* in its French introduced range. More specifically, the aim was to identify and evaluate the genetic links among infestations to identify the invasion pathways that the termite may have followed at national and regional scales. We focused on two highly infested regions, Île-de-France (center = Paris) and Centre-Val de Loire (center = Tours), and estimated population variation, maternal lineages (i.e., haplotypes), and population genetic structure (i.e., microsatellite genotypic distribution). We also characterized colony breeding system along the invasion front in Centre-Val de Loire, from west to northeast. This study contributes to our understanding of how *R. flavipes* spreads within new environments, and confirms that certain anthropogenic factors might strongly affect the termite’s population dynamics.

## 2. Materials and Methods 

### 2.1. Sample Collection

#### 2.1.1. Sampling at the National Scale

To investigate invasive populations of *R. flavipes*, we used specimens from different parts of France (64 sampling locations) and the US (20 sampling locations) collected over the course of a previous study [17] (Table S1).

#### 2.1.2. Sampling at the Regional Scale

Termite workers were collected in two French populations of *R. flavipes*. A first one was sampled in Île-de-France (IDF), a region including the city of Paris, and a second one was sampled 150 km away from Paris, in Centre-Val de Loire (CVL), a region including the cities of Tours and Orléans. Samples were taken from wood fragments, damaged wooden structures, and/or pitfall traps using artificial feeding stations (CVL: 2012 and 2013; IDF: 2014 to 2016). Specimens were stored in 96% ethanol at 4 °C until DNA extraction. Forty-five locations were sampled in IDF (PP1 to PP45), and 62 locations were sampled in CVL (TC01 to TC62). In total, samples were obtained from 36 cities (Table S1). 

#### 2.1.3. Sampling at the Local Scale

In CVL, additional sampling took place at 33 points within the three most infested urban areas—the cities of Tours (15 points: TCT01 to TCT15), Joué-les-Tours (11 points: TCJT01 to TCJT11), and La Riche (7 points: TCLR01 to TCLR07) (Table S2). These 33 samples were used to infer colony breeding structure and social organization within CVL, as these features have already been characterized for IDF [31].

### 2.2. Molecular Procedures

A 658-bp fragment of the mitochondrial cytochrome oxidase II (COII) gene was amplified and sequenced for 107 individuals (45 IDF + 62 CVL) using the modified primers A-tLeu (5’-CAGATAAGTGC-ATTG GATTT-3’) [33] and TK-N-3785 (5’-GTT TAA GAG ACC AGT ACT TG -3’) [34]. PCR amplification was performed using a Multiplex PCR Kit (Qiagen, Valencia, CA, USA) in accordance with the manufacturer’s instructions. PCR templates were sequenced by Genoscreen (Lille, France) using BigDye v. 3.1 and a 96-capillary Applied Biosystems 3730xl DNA Analyzer (Applied Biosystems, Foster City, CA, USA). COII sequence alignment and performance were examined with Geneious v.9.4 (https://www.geneious.com) (Aarhus, Denmark). New sequences were deposited in GenBank under the accession numbers provided in Table S1. 

DNA from individual specimens was extracted using the Wizard^®^ Genomic Purification Kit (Promega, Madison, Wisconsin, USA). To investigate dispersal dynamics at national and regional scales, 100 termites (one per colony; 45 for IDF and 55 of 62 for CVL; 7 individuals were not amplified) were genotyped at the eight highly polymorphic microsatellite loci targeted by the multiplex sets developed by Baudouin et al. 2017 [31]: *Rf1-3*, *Rf6-1*, *Rf15-2*, *Rf21-1*, *Rf11-1*, *RS1*, *RS15*, and *RS43*, which were all previously described by Vargo [35], Dronnet et al. [36], and DeHeer et al. [37]. At the local scale, 660 workers (20 workers from each of the 33 collection points) were genotyped using the 10 highly polymorphic microsatellite loci used in Perdereau et al. [17]: *Rf6-1*, *RS1*, *Rf11-1*, *Rf-21-1*, *RS43*, *Rf24-2*, *RS15*, *Rf1-3*, *RS76*, and *Rf15-2*. PCR amplification and genotyping were performed at Genoscreen platform (http://www.genoscreen.fr/fr/) (Lille, France). Alleles were scored using Geneious v. 9.4.

### 2.3. Genetic Data Analyses at the National Scale

#### 2.3.1. Phylogeographical Analyses

Phylogenetic analyses were performed using four methods: maximum parsimony (MP), neighbor-joining (NJ), maximum-likelihood (ML), and Bayesian inference (BI). The MP and NJ methods were applied using SEAVIEW [38], and the ML method was applied using PHYML v. 3.0 [39]. MRAIC was used to find an appropriate sequence evolution model for the data [40]. BI was carried out using MRBAYES v. 3.2.1 [41], which was run for 5,000,000 generations. No a priori assumptions were made about tree topology, and analyses were carried out using uniform priors. In addition to the 107 COII sequences obtained, we included 84 mitochondrial data for the sampling locations mentioned above from different part of France and US [17]; the same nomenclature was used for the sake of comparison (Table S1). The relationships among haplotypes at the national scale were represented using a haplotype network obtained using TCS v. 1.21 [42]. We used TCSBU software, a web-based program (http://cibio.up.pt/software/tcsBU/), which speeds up the production of publication-ready networks resulting from TCS analysis [43].

#### 2.3.2. Microsatellite and Mitochondrial Data Analyses

To look for evidence of spatial and genetic structure in populations, we analyzed the microsatellite and mitochondrial data (45 points from IDF, 55 points from CVL and 64 points from different parts of France [17]) using Bayesian clustering algorithms implemented in STRUCTURE v. 2.3.4 [44]. This software infers population structure by estimating the number of clusters present (K) and probabilistically assigns individuals to clusters based on the multilocus genotype data. Both of the algorithms assume that clusters are panmictic units with distinct allele frequencies. In STRUCTURE, each run consisted of a burn-in period (length = 10,000) that was followed by 100,000 MCMC simulations using the admixture model. To infer its most likely value, K varied from 1 to 20 across 10 independent runs. The optimal value of K was calculated using the ΔK methods [45] in STRUCTURE HARVESTER v. 0.6 [46]. 

#### 2.3.3. Detection of Bottlenecks

To detect recent bottlenecks, we estimated the deviation between observed heterozygosity and expected heterozygosity under mutation-drift balance using BOTTLENECK v. 1.2.02 [47]. Tests were performed using the genetic clusters previously obtained by STRUCTURE. We used the infinite allele model (IAM), the stepwise mutation model (SMM), and the two-phase model (TPM) (10,000 permutations), and we tested for result significance using the sign test and the Wilcoxon signed-rank test (two-tailed). We also assessed whether there was a mode shift in the allele frequency distribution as an additional sign of a recent genetic bottleneck [47].

### 2.4. Genetic Diversity Measures

Haplotype diversity (*Hd*) and nucleotide diversity (*Nd*) were calculated from the mitochondrial data using ARLEQUIN v. 3.0 [48]. The microsatellite data were used to estimate the number of alleles per locus (*Na*), allelic richness (*Rs*), and gene diversity (*Hs*); Fstat v. 2.9.3 was employed [49]. The significance of any differences between populations in the latter metrics was determined using Kruskal–Wallis tests implemented in the stats package v. 3.3.1 in R (R Development Core team 2015). Genepop on the Web [50] was used to test for the presence of deviation from Hardy–Weinberg equilibrium in the samples as well as for genotypic disequilibrium.

### 2.5. Genetic Data Analyses at the Local Scale

#### 2.5.1. Colony Assignment 

Microsatellite analyses were carried out to determine whether different sampling locations were associated with the same colony. Genotype frequencies were compared for all pairs of locations using a log-likelihood (G)-based differentiation test from Genepop on the Web. Overall significance was determined using Fisher’s combined probability test, with a Bonferroni correction for multiple comparisons. Samples taken from two different locations were considered to belong to different colonies if their genotypic differences were statistically significant [27,30,51]. 

#### 2.5.2. Colony Breeding Structure

The breeding structure of each colony was determined using Genepop on the Web, which allows colonies to be classified according to family type (i.e., simple, extended, and mixed). To do this, the observed number and frequency of alleles and genotypes within colonies were compared to expected values for the three family types [27,35,52]. Colonies were classified as simple families when genotype values were consistent with those expected for the direct offspring of a single pair of reproductives and when the observed frequencies did not differ significantly from those expected under Mendelian segregation of alleles from two parents. Significance was determined by a G-test (*p* < 0.05) carried out across all loci. Colonies were classified as extended families when there were four or fewer alleles at all loci, and when worker genotypes were not consistent with those expected for a single pair of reproductives (e.g., more than four genotypes at a locus or three or more homozygote genotypes) or when genotype frequencies deviated significantly from those expected for simple families. Colonies were classified as mixed families when more than four alleles were found at one or more loci, a pattern that is consistent with offspring produced by more than two unrelated reproductives.

#### 2.5.3. Isolation by Distance

Isolation by distance (i.e., a positive correlation between geographical distance and genetic differentiation) was calculated for all the sampling locations for each site (Tours, Joué-les-Tours, and La Riche). The correlation coefficient between *F_ST_*/(1-*F_ST_*) and the natural-log-transformed geographical distances between sampling locations [53] was obtained by using the Mantel test in Genepop on the Web.

## 3. Results

### 3.1. National and Regional Invasion Patterns

The alignment of the 191 COII sequences revealed 44 variable positions along the 658-bp sequence, which resulted in a total of 18 haplotypes. In France, there were twelve haplotypes, which were named A, B, C, D, E, F, G, H, I, J, K, and CE (newly identified).

**Figure 1:**
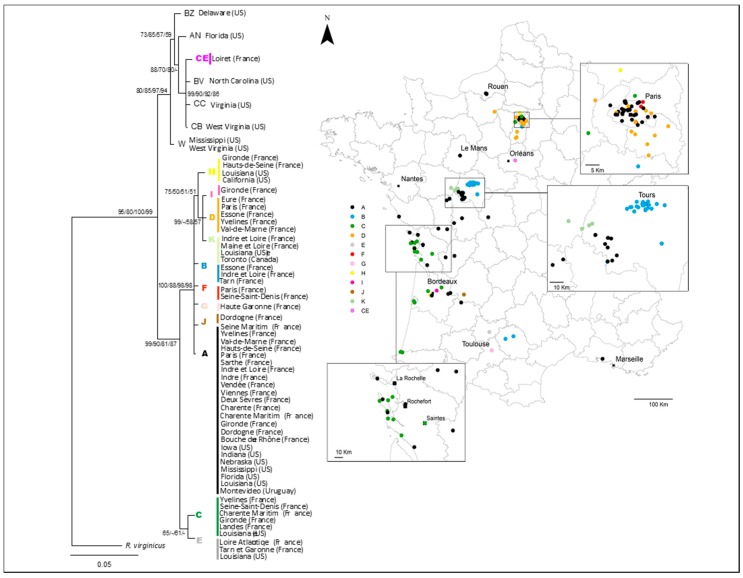
Phylogenetic tree of *Reticulitermes flavipes* mtDNA haplotypes obtained using Bayesian inference. Haplotypes are indicated by colored letters and dots. The map shows the geographical distribution of the samples.

The phylogenetic trees constructed by the four methods (MP, NJ, ML, and BI) arrived at congruent topologies and confirmed that the samples fell into two main clades, as previously observed [17] (Figure 1). One clade was composed of haplotype CE (CVL, near Orléans) and 6 haplotypes from the US Atlantic Coast. The second clade was composed of the 11 remaining haplotypes present in France, which was divided into two subclades: one with the haplotypes A, B, D, F, G, H, I, J, and K and the second with the haplotypes C and E. Seven haplotypes were exclusively found in France (B, D, F, G, I, J, and CE) and 5 haplotypes (A, C, E, H, and K) also occurred in the Louisianan source population.

**Figure 2:**
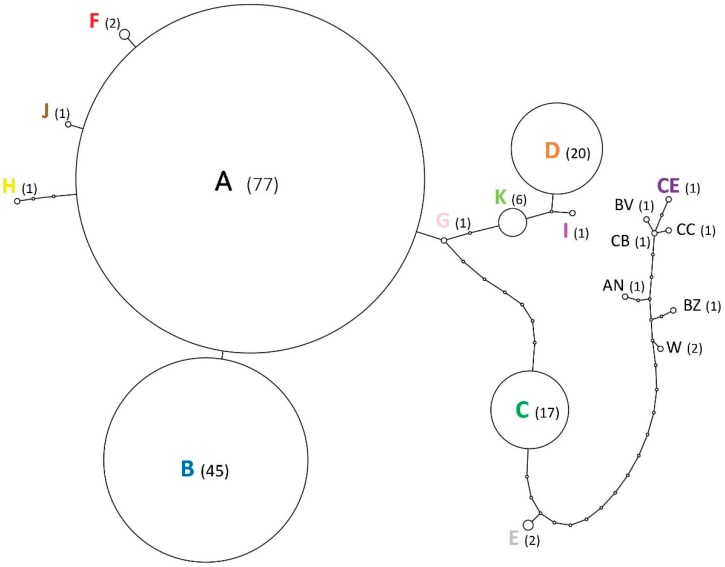
Minimum-spanning network of *Reticulitermes flavipes* mtDNA haplotypes. The haplotypes are color coded, and sample size is indicated in parentheses.

Parsimony network analysis revealed the relationship among the 18 COII haplotypes given a 90% confidence interval (Figure 2). Haplotypes diverged by 1 to 19 mutational steps; the newly identified haplotype CE was the most divergent. The dominant haplotype in France is haplotype A, which was represented in 45% of French samples. This haplotype was found across the entirety of introduced range studied here. The results for the COII haplotypes revealed that eight haplotypes (B, D, F, G, H, I, J, and K) diverged from haplotype A by one to four mutational steps. Haplotype B (represented in 26% of French samples) predominated in CVL; it also occurred a few times in IDF and in southwestern France. Haplotype D, which was represented in 12% of French samples, was only observed in IDF. Haplotype C (represented in 10% of French samples) diverged from haplotype A by seven mutational steps and was seen along France’s western coast and in IDF. Haplotype E diverged from haplotype C by three mutational steps. The remaining haplotypes (F, G, H, I, J, and K) were each represented by just a few samples.

Focusing on the regional scale, we noted that the dominant haplotype in IDF was the predominant haplotype in France, haplotype A, whereas haplotype B was the most common haplotype in CVL. However, both haplotypes occurred in both IDF and CVL. The two study regions differed in the spatial distribution of mitochondrial variants—the pattern was heterogeneous in IDF, while single-haplotype clusters were seen in CVL. IDF and CVL haplotype numbers were 6 and 4, respectively, and the haplotype diversity indices were 0.561 and 0.531, respectively (Table S3). In Charente-Maritime, two haplotypes—A and C—were observed. It is worth noting, however, that the main haplotype, A, was present in both La Rochelle and Rochefort.

### 3.2. Relationships among Infestations

**Figure 3:**
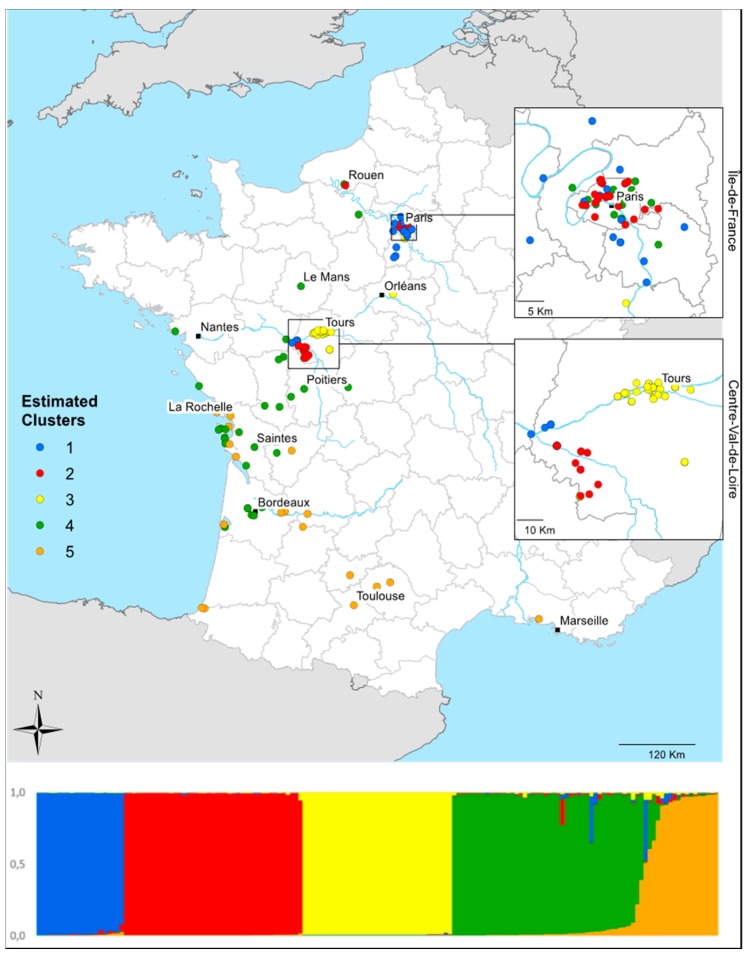
Map of *Reticulitermes flavipes* sampling locations in France. The map was created using ArcMap (ArcGIS v. 10.3.1). At the bottom of the figure, the assignment probability in the five clusters identified by STRUCTURE based on eight microsatellite loci is represented for each of 164 individuals: S1 (blue; n = 21), S2 (red; n = 43), S3 (yellow; n = 36), S4 (green; n = 46), and S5 (orange; n = 18).

The STRUCTURE analysis revealed that the 164 colonies in France (IDF = 45, CVL = 55, and 64 from Perdereau et al. 2013 [17]) were grouped into five genetic clusters (K = 5, numbered S1 to S5) (Figure 3). In the S1 cluster (blue) were 21 samples that mostly came from IDF but also from the western edge of CVL. The S2 cluster (red) contained 43 samples from IDF, mostly from Paris and the western edge of CVL, but also from one location next to Rouen. In the S3 cluster (yellow) were 36 samples from in and around Tours, 1 sample from near Orléans, and 1 sample from IDF. In the S4 cluster (green) were 46 samples from the western coast and the northern half of the country (16 samples from IDF) as well as from a single location next to Rouen. Finally, the S5 cluster (orange) contained 18 samples collected along the western coast and the southern half of the country, including a single location near Marseille.

### 3.3. Genetic Diversity and Bottlenecks

The microsatellite analyses showed that the clusters displayed significant genetic differentiation (Table S4). None of the clusters (S1 to S5) deviated from Hardy–Weinberg equilibrium (*p* > 0.05). One genotypic linkage disequilibrium was observed: between loci Rf11-1 and Rf15-2 in cluster S1. The STRUCTURE genetic analyses showed that allele number per locus (Na), allelic richness (Rs), and gene diversity (Hs) were significantly lower in S3 than in the other four clusters (i.e., mean Na: 2.25, Rs = 2.06 and Hs = 0.257; Kruskal–Wallis test: *p* < 0.05) (Table 1). S4 and S5 had higher numbers of alleles per locus (Na) and allelic richness (Rs) than did the other three clusters (Table 1).

Recent bottlenecks were evidenced in S1, S2, and S4 (Table 2). For S1, statistical support for a significant bottleneck came from IAM (one-tailed Wilcoxon signed-rank test: *p*-value = 0.027). In the case of S2, support came from both IAM (sign test: *p*-value = 0.005; one-tailed and two-tailed Wilcoxon signed-rank tests: *p*-values = 0.002 and 0.004, respectively) and TPM (one-tailed Wilcoxon signed-rank test: *p*-value = 0.037). For S4, support came from the SMM (two-tailed sign and Wilcoxon signed-rank tests: *p*-values = 0.0007 and 0.0039, respectively). No mode shifts in allele frequency distribution were observed in any of the clusters.

### 3.4. Local Invasion Patterns in Centre-Val de Loire

At the local scale, in CVL, only six loci were included in the analyses because four loci were found to be monomorphic (RS43, Rf1-3, RS76, and Rf15-2). For these six microsatellite loci, there were an average of 2.3 alleles per locus (range 2–3) (Table 3). There was no significant difference in allelic richness (R_S_) and gene diversity (H_S_) among sites (Table 3). There was no genotypic disequilibrium at any loci. Genotype differentiation tests grouped the 15 samples collected in Tours into a single colony. Similarly, the 11 samples taken in Joué-les-Tours also belong to a same colony, and the 7 sampling locations in La Riche into a third colony (G-test values between pairs of sampling locations: Tours: *p* > 0.0003, Joué-les-Tours: *p* > 0.0004, and La Riche: *p* > 0.0007). Based on the number of genotypes, these three colonies were classified as extended families (i.e., colonies containing neotenics). Because colonies displayed a small number of alleles per locus, it was impossible to determine whether it was actually a mixed family as opposed to an extended family [28]. There was no significant isolation by distance within the three colonies (Mantel tests: n = 15, r^2^ = 0.001, *p* = 0.348 for Tours; n = 11, r^2^ = 0.003, *p* = 0.024 for Joué-les-Tours; and n = 7, r^2^ = 0.053, *p* = 0.261 for La Riche).

## 4. Discussion

### 4.1. Spread of Reticulitermes flavipes Across France

It has been hypothesized that *R. flavipes* was first introduced to one or several ports (Rochefort and La Rochelle; Charente-Maritime) along the French Atlantic Coast, arriving on ships coming from Louisiana during the XVIII century. According to this scenario, introduced populations would have dispersed to other cities and forests. This hypothesis is supported by the genetic results obtained in this study. The current haplotype distribution in France suggests that a limited number of propagules containing only two haplotypes, A and C, initially arrived in Charente-Maritime. In addition, several punctual introduction events could have occurred independently into Gironde, Paris, Maine et Loire, Loire Atlantic, Tarn et Garonne and Loiret counties which present four haplotypes H, K, E and CE also found in US.

Several haplotypes identified in France diverged by only a few mutations from haplotypes A and C. We found that the predominant haplotype in France (haplotype A) was present in Rochefort and La Rochelle, two major port cities that played an important role in international trades when Louisiana was a colonial territory belonging to the kingdom of France [20,21]. Based on the haplotype distribution pattern and the STRUCTURE results (Figure 1 and Figure 2), it would appear that these first invasive populations then spread within Charente-Maritime, along the Atlantic Coast, and toward Northeastern and Southeastern France. The results obtained using the microsatellite and mitochondrial data suggest that there was expansion along two major fronts. In the case of the first front, *R. flavipes* appears to have spread toward Southeastern France, infesting the Saintonge region (Poitou-Charente, represented by the city of Saintes) and then the region of Bordeaux before reaching southern France (Toulouse and Martigues). In the case of the second front, *R. flavipes* seems to have headed toward Northeastern France, with a series of introductions taking place within the Poitevin region (city of Poitiers) and Touraine region (Tours), before the termite reached Paris and more distant northern cities (Rouen and Orléans). Along these expansion fronts, new variant haplotypes (F, J, B, G, D and I) could have diverged from the haplotypes of the initial invasive populations (A and C). As for haplotype CE, which is highly distinct from the other haplotypes found in France, it has only been observed in the Loiret region (next to city of Orléans). It is closely related to a variant found in North Carolina and Virginia in the US. With our data, the most parsimonious hypothesis to explain the presence of this divergent haplotype in France is an independent introduction event. 

### 4.2. Evidence of Population Bottlenecks

It is well known that populations of introduced species often experience genetic bottlenecks [2,54]. In this study, however, evidence for population bottlenecks following the introduction of *R. flavipes* into France is unclear. The partial support for such events in certain genetic clusters could be due to a deficit in heterozygosity, which is often seen in introduced populations of *R. flavipes* and can result from inbreeding among related neotenics [29]. In addition, a number of generations have passed since the introduction event, which means signs of bottleneck could have diminished. That said, a loss in genetic diversity was observed within the front expanding toward northern France (Table 1). Consequently, it appears that these invasive populations experienced a reduction in genetic diversity in relation to long-distance dispersal events during post-establishment expansion.

### 4.3. Termite Spread Driven by the Railway Network 

**Figure 4:**
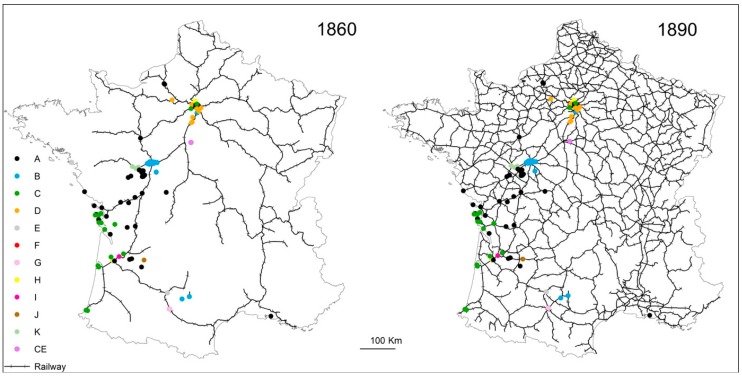
Development of the French railway network over time. The maps show the state of the network in the 1860s and 1890s and the haplotypes (colored letters and dots) at each *Reticulitermes flavipes* sampling location (free access mapping sources: SNCF Réseau, https://data.sncf.com and EuroGeographic, https://eurogeographics.org/products-and-services/open-data).

The dynamics behind the expansion of *R. flavipes* in France have long been an open question. Numerous studies have underscored the role played by human-mediated transport, which has facilitated the insects’s long-distance dispersal and increased its rate of spread [10,12,13,14]. Subterranean termites can establish themselves and spread within cities, which provide hospitable habitat [31]. Study has also recently shown that termite infestations in Paris may spread via railway transport, a pattern that was also observed in CVL [32,55,56]. It should be noted that, in several regions, termite infestations have often been found in forests in the vicinity of railroad tracks (personal communication). The construction of France’s railroad network began in 1847 in Paris (St Lazare train station). It quickly expanded beyond Paris to other parts of the country, and its current structure was largely put into place over the 50 years that followed (Figure 4). 

It may have been fully or nearly concomitant with the arrival of *R. flavipes* in France, which is thought to have occurred in the 18th century on the Atlantic Coast and in the Saintonge region; the train reached both rather early on (i.e., before 1860 [57]). We may think that Paris has been quickly infested by *R. flavipes*, even if the first record of its presence only arrived in Paris dates back to 1922 [58]. In CVL, *R. flavipes* was first observed in 1965 in Chouzé-sur-Loire; records in and around Tours are more recent (1980s) [32,55]. Some colonies in CVL have been found less than 200 meters from railway tracks, which means that they might be connected to other colonies in this area, to the north (i.e., Paris), and along the Atlantic Coast, where termite infestations are more widespread and denser. Indeed, the spread of the termite over long distances seems to have been largely facilitated by the railway network in France (Figure 4), as it has been observed in *Coptotermes* species [59]. Several studies have effectively shown that railroads are a major dispersal mode of the highly invasive termite *Coptotermes formosanus* in southern US [60,61,62]. It has been also noticed that infestations are often associated with recycled railroad ties used in building construction and landscaping [62]. Altogether, results of this study support the hypothesis that successive introductions took place from the Atlantic Coast, step by step, from one city to the next, to the north and to the south of France via railway network.

### 4.4. Regional-Scale Invasion Patterns 

The analyses looking at phylogeography and genetic structure revealed the presence of different distribution patterns within the two focal study regions. In CVL, the distribution of infestations seems rather aggregative. Except for the termites taken from near Orléans (which displayed the unique haplotype CE), all the other aggregates were characterized by a single haplotype and formed genetic clusters; this result suggests that there were three termite outbreaks that locally expanded over the years following the initial introduction events. In contrast, the distribution pattern was highly heterogenous in IDF. The genetic clusters identified by STRUCTURE were not continuous units, as would be expected if populations were structured. Eighty percent of the clusters identified in the French populations were present in IDF (Figure 3). The STRUCTURE analysis likely reflects that the individuals sampled have a recent common origin, but this interpretation must be made with caution because the results could be due to a founder effect and/or genetic drift [63,64]. We think that, in Paris and IDF, several independent introduction events occurred and that little gene flow took place among local infestations. In large cities such as Paris, many human activities including movement along lines of transport and global trade, may favor termite introductions and dispersion. Another interesting result is that three of the genetic clusters within IDF were also observed in CVL, which suggests that some clusters may have been derived from others. Indeed, given the genetic diversity of the clusters within both regions, the Paris or IDF infestations could be the source of the CVL infestations.

### 4.5. Local-Scale Invasion Patterns

Previous research has shown that all introduced populations of *R. flavipes* exhibit a particular colony breeding structure, in which hundreds of neotenics are present and colonies have a propensity to fuse, traits that occur in both forests (i.e., Olonnes, Oléron) and urban populations (i.e., Paris) [28,29,30,31]. These two characteristics may have allowed introduced populations of *R. flavipes* to successfully invade new areas [29]. Local-scale genetic results in CVL revealed that each area formed a single populous colony that encompassed several sampling locations. Each colony appeared to be extremely large, extending over at least 3500 m^2^ for Tours; 7600 m^2^ for Joué-Les-Tours; and 2600 m^2^ for La Riche. In introduced populations of *R. flavipes*, colonies are often large in both forests and urban areas [29]. These three colonies contained several secondary reproductives, which are known to contribute to colony budding and spatial coverage [29]. Unfortunately, the limited number of colonies observed at the local scale in CVL did not permit to estimate the number of neotenics within colonies using F-statistics. Furthermore, the populations exhibited the lowest mean number of alleles (2.33 ± 0.52, Table 3) never detected to date across all *R. flavipes* populations [29,31], making it impossible to detect colony fusion in this population [28].

## 5. Conclusions

This study provides an evidence-supported scenario for how *R. flavipes*, a termite species that is a major pest, invaded and spread throughout France after its introduction about two centuries ago. In a previous study, we identified the putative source population of French populations [17]; these new findings represent a second step in efforts to reconstruct the termite’s invasion dynamics. They also highlight the role played by human-mediated long-distance transport in the termite’s dispersal, which seems to have been facilitated by the French railway network. This study will improve understanding of the termite’s history and invasion process and could help inform control strategies.

## Figures and Tables

**Table 1:** Genetic diversity of the five *Reticulitermes flavipes* clusters (S1 to S5) identified by Structure using eight microsatellite loci (n = 164 workers). The number of alleles per locus (*Na*), allelic richness (*Rs*), and gene diversity (*Hs*) were calculated.

Loci	The Number of Alleles					
	***Na***					
**Locus**	**S1**	**S2**	**S3**	**S4**	**S5**	**All**
***Rf11-1***	4	6	4	6	7	10
***Rf6-1***	8	7	4	11	11	20
***Rs1***	5	5	2	15	10	19
***Rf21-1***	7	6	2	24	18	32
***Rs43***	3	2	1	4	4	5
***Rf15-2***	3	3	1	4	5	5
***Rs15***	3	4	3	19	7	21
***Rf1-3***	6	2	1	10	4	10
**Mean**	4.88	4.38	2.25	11.63	8.25	15.25
**± SD**	±1.96 (a)	±1.92 (a)	±1.28 (b)	±7.27 (c)	±4.71 (a,c)	±9.38
						
	***Rs***					
**Locus**	**S1**	**S2**	**S3**	**S4**	**S5**	**All**
***Rf11-1***	4	4.67	3.94	4.45	7	7.44
***Rf6-1***	7.98	5.31	3.50	8.98	11	13.06
***Rs1***	4.84	4.81	1.50	11.80	10	10.79
***Rf21-1***	6.89	5.05	2	17.29	18	16.62
***Rs43***	2.8§	2	1	2.68	4	3.83
***Rf15-2***	3	2.96	1	3.43	5	4.90
***Rs15***	2.98	3.90	2.55	14.17	7	10.86
***Rf1-3***	5.97	2	1	8.95	4	6.95
**Mean**	4.81	3.84	2.06	8.97	8.25	9.31
**± SD**	±1.96 (a,c)	±1.35 (a)	±1.17 (b)	±5.28 (a,c)	±4.71 (c)	±4.32
						
	***Hs***					
**Locus**	**S1**	**S2**	**S3**	**S4**	**S5**	**All**
***Rf11-1***	0.727	0.587	0.612	0.591	0.837	0.670
***Rf6-1***	0.867	0.711	0.671	0.852	0.895	0.799
***Rs1***	0.564	0.767	0.028	0.884	0.828	0.614
***Rf21-1***	0.782	0.648	0.44	0.944	0.951	0.752
***Rs43***	0.331	0.506	0	0.115	0.413	0.272
***Rf15-2***	0.612	0.542	0	0.525	0.716	0.477
***Rs15***	0.423	0.654	0.307	0.914	0.704	0.601
***Rf1-3***	0.669	0.307	0	0.835	0.503	0.463
**Mean**	0.622 (a)	0.590 (a)	0.257 (b)	0.707 (a)	0.731 (a)	0.581

The letters indicate that significant differences were present between clusters (Kruskal–Wallis tests, nonparametric ANOVA: *Na* χ^2^ = 19.5, d.f. = 4, *p* < 0.001; *Rs* χ^2^ = 19.4, d.f. = 4, *p* < 0.001; *Hs* χ^2^ = 12.9, d.f. = 4, *p* < 0.05.

**Table 2:** Results of the statistical tests examining evidence for recent bottlenecks in the five *Reticulitermes flavipes* genetic clusters identified by Structure.

	Sign Test			Wilcoxon Test						Mode-Shift
				One Tail			Two Tails			
	IAM	TPM	SMM	IAM	TPM	SMM	IAM	TPM	SMM	
**S1**	NS	NS	NS	*p* < 0.05	NS	NS	NS	NS	NS	Normal
**S2**	*p* < 0.01	NS	NS	*p* < 0.01	*p* < 0.05	NS	*p* < 0.01	NS	NS	Normal
**S3**	NS	NS	NS	NS	NS	NS	NS	NS	NS	Normal
**S4**	NS	NS	*p* < 0.001	NS	NS	NS	NS	NS	*p* < 0.01	Normal
**S5**	NS	NS	NS	NS	NS	NS	NS	NS	NS	Normal

Significant results indicate that there was an excess of heterozygotes in the cluster. NS = not significant; IAM: infinite allele model; TPM: two-phase model; SMM: stepwise mutation model.

**Table 3:** Variability at six microsatellite loci for *Reticulitermes flavipes* termites taken from colonies in and around Tours, France. The number of alleles per locus (*Na*), allelic richness (*Rs*), and gene diversity (*Hs*) were calculated using all the samples.

			Tours			Joué-Les-Tours			La Riche	
**Locus**	***Na***		***Rs***	***Hs***		***Rs***	***Hs***		***Rs***	***Hs***
***Rf6-1***	3		2.55	0.561		2.60	0.590		2.88	0.643
***RS1***	2		1.42	0.149		1.20	0.062		1.90	0.335
***Rf11-1***	3		2.41	0.557		2.72	0.683		2.58	0.502
***Rf21-1***	2		1.85	0.380		1.77	0.349		1.86	0.306
***Rf24-2***	2		1.93	0.474		1.94	0.469		1.99	0.472
***RS15***	2		1.59	0.242		1.70	0.241		1.94	0.359
**Mean **	2.33		1.96	0.394		1.99	0.399		2.19	0.436
**± SD**	±0.52		±0.44 (a)			±0.58 (a)			±0.43 (a)	

The letters inside the parentheses indicate significant differences between colonies (Kruskal–Wallis tests, nonparametric ANOVA, *Rs* χ^2^ = 1.72, d.f. = 2, *p* > 0.05; *Hs* χ^2^ = 0.082, d.f. = 2, *p* > 0.05).

## Supplementary Materials

The following are available online at http://www.mdpi.com/2075-4450/10/1/30/s1, Table S1: Description of the *Reticulitermes flavipes* samples: sampling location, sampling region, GPS coordinates (latitude/longitude), GenBank accession number, mtDNA COII haplotype, samples genotyped using microsatellites, and genetic cluster membership (based on STRUCTURE analysis of eight microsatellite loci for termite workers collected for this study in Centre-Val de Loire (CVL) and Île-de-France (IDF) and as part of Perdereau et al. [17]), Table S2: Description of *Reticulitermes flavipes* samples collected from in and around Tours: sampling locations and GPS coordinates (latitude/longitude), Table S3: Haplotype diversity of the mitochondrial gene COII in *Reticulitermes flavipes* in France, Table S4: Genetic differentiation among the five *Reticulitermes flavipes* clusters in France identified by STRUCTURE using eight microsatellite loci (n = 164 workers). Pairwise population *F*_ST_ values were obtained after 1000 permutations.
