# Supplementary material for: Invasion Dynamics of A Termite, Reticulitermes flavipes, at Different Spatial Scales in France
Source: Insects. 2019 Jan 15;10(1):30. doi: 10.3390/insects10010030 (PMC6358928; doi:10.3390/insects10010030)
Supplement: Supplementary file 1 [file insects-10-00030-s001.pdf]

Table S1. Description of the *Reticulitermes flavipes* samples: sampling location, sampling region, GPS coordinates (latitude/longitude), GenBank accession number, mtDNA COII haplotype, samples genotyped using microsatellites, and genetic cluster membership (based on STRUCTURE analysis of eight microsatellite loci for termite workers collected for this study in Centre-Val de Loire [CVL] and Île-de-France [IDF] and as part of Perdereau *et al.* [17]).

Sample ID	Location	Region	GPS coordinates		GenBank Accession Number	mtDNA COII Haplotype	Genotyped Microsatellites	STRUCTURE Cluster
			Lat	Lon				
TC01	Joué-lès-Tours	CVL	0.672386	47.352804	MF447379	B	X	S3
TC02	Joué-lès-Tours	CVL	0.672552	47.352256	MF447380	B	X	S3
TC03	Richelieu	CVL	0.318788	47.012432	MF447381	A	X	S2
TC04	Richelieu	CVL	0.361836	47.018821	MF447382	A	X	S2
TC05	Tours	CVL	0.668824	47.385258	MF447383	B	X	S3
TC06	Chouzé sur Loire	CVL	0.126416	47.238239	MF447384	K	X	S1
TC07	Chouzé sur Loire	CVL	0.057419	47.214058	MF447385	K	X	S1
TC08	Chouzé sur Loire	CVL	0.057418	47.214058	MF447386	K	-	-
TC09	La Ville aux Dames	CVL	0.780485	47.389017	MF447387	B	X	S3
TC10	La Ville aux Dames	CVL	0.780304	47.389065	MF447388	B	X	S3
TC11	St Genouph	CVL	0.601660	47.364034	MF447389	B	-	-
TC12	La Riche	CVL	0.653515	47.378117	MF447390	B	X	S3
TC13	Berthenay	CVL	0.523843	47.363269	MF447391	B	X	S3
TC14	Fondettes	CVL	0.645456	47.398418	MF447392	B	X	S3
TC15	Saint Avertin	CVL	0.730555	47.357361	MF447393	B	X	S3
TC16	Tours	CVL	0.704274	47.380846	MF447394	B	X	S3
TC17	Lemeré	CVL	0.313972	47.100947	MF447395	A	X	S2
TC18	La Ville aux Dames	CVL	0.780485	47.389017	MF447396	B	X	S3
TC19	St Genouph	CVL	0.584546	47.369234	MF447397	B	X	S3
TC20	Berthenay	CVL	0.534227	47.364001	MF447398	B	X	S3
TC21	Montlouis sur Loire	CVL	0.859446	47.381838	MF447399	B	X	S3
TC22	La Riche	CVL	0.651186	47.377883	MF447400	B	X	S3
TC23	Tours	CVL	0.693902	47.360997	MF447401	B	X	S3

Sample ID	Location	Region	GPS coordinates		GenBank Accession Number	mtDNA COII Haplotype	Genotyped Microsatellites	STRUCTURE Cluster
			Lat	Lon				
TC24	Manthelan	CVL	0.839098	47.136961	MF447402	B	X	S3
TC25	Cravant les Coteaux	CVL	0.345702	47.158618	MF447403	A	X	S2
TC26	Chaveignes	CVL	0.405129	47.052326	MF447404	A	X	S2
TC27	Savonnières	CVL	0.561769	47.336450	MF447405	B	-	-
TC28	Savonnières	CVL	0.561572	47.336345	MF447406	B	X	S3
TC29	La Riche	CVL	0.648383	47.385851	MF447407	B	X	S3
TC30	La Riche	CVL	0.648383	47.385851	MF447408	B	X	S3
TC31	Berthenay	CVL	0.490155	47.351929	MF447409	B	X	S3
TC32	Berthenay	CVL	0.490155	47.351929	MF447410	B	X	S3
TC33	Joué-lès-Tours	CVL	0.671869	47.346523	MF447411	B	X	S3
TC34	Beaumont en Véron	CVL	0.189572	47.178468	MF447412	A	X	S2
TC35	Cravant les Coteaux	CVL	0.312551	47.162484	MF447413	A	X	S2
TC36	Cravant les Coteaux	CVL	0.313507	47.162481	MF447414	A	X	S2
TC37	Cravant les Coteaux	CVL	0.314891	47.162313	MF447415	A	-	-
TC38	Savonnières	CVL	0.561769	47.336450	MF447416	B	X	S3
TC39	Fay aux loges	CVL	2.144386	47.923287	MF447417	CE	X	S3
TC40	Berthenay	CVL	0.523818	47.363357	MF447418	B	X	S3
TC41	La Riche	CVL	0.660084	47.380681	MF447419	B	-	-
TC42	La Riche	CVL	0.642519	47.378285	MF447420	B	X	S3
TC43	La Riche	CVL	0.638916	47.387371	MF447421	B	X	S3
TC44	Berthenay	CVL	0.524826	47.362359	MF447422	B	-	-
TC45	Fondettes	CVL	0.583159	47.398897	MF447423	B	X	S3
TC46	Tours	CVL	0.695069	47.402991	MF447424	B	X	S3
TC47	Manthelan	CVL	0.838949	47.137636	MF447425	B	-	-
TC48	Manthelan	CVL	0.839400	47.137772	MF447426	B	X	S3
TC49	Beaumont en Véron	CVL	0.190431	47.178306	MF447427	A	X	S2
TC50	Beaumont en Véron	CVL	0.189979	47.178763	MF447428	A	X	S2

Sample ID	Location	Region	GPS coordinates		GenBank Accession Number	mtDNA COII Haplotype	Genotyped Microsatellites	STRUCTURE Cluster
			Lat	Lon				
TC51	Beaumont en Véron	CVL	0.189782	47.178548	MF447429	A	X	S2
TC52	Beaumont en Véron	CVL	0.190341	47.178579	MF447430	A	X	S2
TC53	Beaumont en Véron	CVL	0.189460	47.177700	MF447431	A	X	S2
TC54	Beaumont en Véron	CVL	0.190146	47.178141	MF447432	A	X	S2
TC55	Chouzé sur Loire	CVL	0.155466	47.249421	MF447433	K	X	S1
TC56	Chouzé sur Loire	CVL	0.155466	47.249421	MF447434	K	X	S1
TC57	Ligré	CVL	0.287359	47.122481	MF447435	A	X	S2
TC58	Berthenay	CVL	0.523357	47.363168	MF447436	B	X	S3
TC59	St Cyr sur Loire	CVL	0.648244	47.396521	MF447437	B	X	S3
TC60	Lemeré	CVL	0.313992	47.100907	MF447438	A	X	S2
TC61	Richelieu	CVL	0.315087	47.009289	MF447439	B	X	S3
TC62	Richelieu	CVL	0.315087	47.009289	MF447440	B	X	S3
PP01	Paris 9th	IDF	2.328885	48.883059	MF447334	D	X	S1
PP02	Paris 8th	IDF	2.309844	48.872253	MF447335	D	X	S1
PP03	Paris 16th	IDF	2.269412	48.862134	MF447336	D	X	S1
PP04	Paris 13th	IDF	2.366112	48.832472	MF447337	D	X	S1
PP05	Paris 16th	IDF	2.273899	48.855622	MF447338	A	X	S2
PP06	Paris 16th	IDF	2.265278	48.856083	MF447339	A	X	S2
PP07	Paris 8th	IDF	2.297101	48.874092	MF447340	A	X	S2
PP08	Paris 8th	IDF	2.305418	48.866640	MF447341	A	X	S2
PP09	Paris 8th	IDF	2.308376	48.869948	MF447342	A	X	S2
PP10	Paris 19th	IDF	2.375387	48.892660	MF447343	A	X	S2
PP11	Paris 17th	IDF	2.316588	48.892582	MF447344	A	X	S2
PP12	Paris 17th	IDF	2.314745	48.895147	MF447345	A	X	S2
PP13	Paris 17th	IDF	2.312607	48.894377	MF447346	A	X	S2
PP14	Paris 4th	IDF	2.358742	48.854871	MF447347	A	X	S2

Sample ID	Location	Region	GPS coordinates		GenBank Accession Number	mtDNA COII Haplotype	Genotyped Microsatellites	STRUCTURE Cluster
			Lat	Lon				
PP15	Paris 19th	IDF	2.383244	48.895670	MF447348	A	X	S2
PP16	Paris 15th	IDF	2.297947	48.836407	MF447349	A	X	S2
PP17	Paris 6th	IDF	2.340149	48.849259	MF447350	A	X	S2
PP18	Paris 2nd	IDF	2.326792	48.869445	MF447351	A	X	S2
PP19	Clichy	IDF	2.310576	48.898353	MF447352	A	X	S2
PP20	Paris 2nd	IDF	2.336417	48.871278	MF447353	A	X	S2
PP21	Paris 8th	IDF	2.325101	48.871073	MF447354	A	X	S2
PP22	Paris 9th	IDF	2.334690	48.872598	MF447355	A	X	S2
PP23	Paris 17th	IDF	2.313111	48.898861	MF447356	A	X	S2
PP24	Paris 13th	IDF	2.376889	48.823810	MF447357	A	X	S2
PP25	Paris 19th	IDF	2.376382	48.893711	MF447358	A	X	S2
PP26	Paris 20th	IDF	2.404611	48.876833	MF447359	A	X	S2
PP27	Paris 12th	IDF	2.398848	48.833127	MF447360	A	X	S2
PP28	Paris 7th	IDF	2.298639	48.860268	MF447361	A	X	S2
PP29	Morigny champigny	IDF	2.345548	48.802870	MF447362	D	X	S1
PP30	Etampes	IDF	2.189168	48.451230	MF447363	D	X	S1
PP31	Kremlin-Bicètre	IDF	2.155279	48.431944	MF447364	D	X	S1
PP32	Sotteville-Lès-Rouen	-	1.081944	49.413889	MF447365	A	X	S2
PP33	Juvisy-Sur-Orge	IDF	2.380556	48.693056	MF447366	B	X	S3
PP34	Puteaux	IDF	2.288889	48.996667	MF447367	H	X	S1
PP35	Etampes	IDF	2.2027778	48.573333	MF447368	D	X	S1
PP36	Chatou	IDF	2.163333	48.900556	MF447369	D	X	S1
PP37	Fontenay sous Bois	IDF	2.461945	48.850833	MF447370	A	X	S2
PP38	Champigny sur Marne	IDF	2.529722	48.820833	MF447371	D	X	S1
PP39	Paris 17th	IDF	2.312791	48.895372	MF447372	A	X	S2
PP40	Villeneuve le roi	IDF	2.432681	48.727928	MF447373	D	X	S1

Sample ID	Location	Region	GPS coordinates		GenBank Accession Number	mtDNA COII Haplotype	Genotyped Microsatellites	STRUCTURE Cluster
			Lat	Lon				
PP41	Villejuif	IDF	2.364362	48.794416	MF447374	D	X	S1
PP42	Villejuif	IDF	2.364602	48.794454	MF447375	D	X	S1
PP43	Choisy le roi	IDF	2.425551	48.763443	MF447376	D	X	S1
PP44	Versailles	IDF	2.130490	48.796663	MF447377	C	X	S1
PP45	Saint Denis	IDF	2.361577	48.916058	MF447378	C	X	S1
Fr01	Petit Quevilly	-	1.05388900	49.43111100	JQ280596	A	X	S4
Fr02	Pacy sur Eure	-	1.38280000	49.01670000	JQ280576	D	X	S4
Fr03	Paris 7th	IDF	2.30026880	48.85758130	JQ280605	A	X	S4
Fr04	Paris 8th	IDF	2.34769810	48.84049180	JQ280604	A	X	S4
Fr05	Paris 8th	IDF	2.32608690	48.87057530	JQ280603	A	X	S4
Fr06	Paris 9th	IDF	2.34567040	48.88236550	JQ280606	A	X	S4
Fr07	Paris 11th	IDF	2.36571720	48.86661990	JQ280607	A	X	S4
Fr08	Paris 11th	IDF	2.36543040	48.86636790	JQ280572	D	X	S4
Fr09	Paris 11th	IDF	2.36822810	48.85693410	JQ280571	A	X	S4
Fr10	Paris 13th	IDF	2.36623980	48.83447990	JQ280577	D	X	S4
Fr11	Paris 13th	IDF	2.36828200	48.83175880	JQ280578	A	X	S4
Fr12	Paris 16th	IDF	2.39252190	48.88214850	JQ280579	F	X	S4
Fr13	Paris 16th	IDF	2.27917550	48.86543140	JQ280580	A	X	S4
Fr14	Puteaux	IDF	2.23888900	48.88500000	JQ280582	A	X	S4
Fr15	Pantin	IDF	2.40168000	48.89658000	JQ280584	F	X	S4
Fr16	Bagnolet	IDF	2.41810000	48.86920000	JQ280581	D	X	S4
Fr17	Montreuil	IDF	2.44361100	48.86111100	JQ280583	D	X	S4
Fr18	Créteil	IDF	2.46277800	48.79111100	JQ280585	D	X	S4
Fr19	Le Mans	-	0.19694400	48.00416700	JQ280575	A	X	S4
Fr20	La Baule-Escoublac	-	-2.39080000	47.28670000	JQ280554	E	X	S4
Fr21	Saumur	-	-0.07694410	47.26000000	JQ280566	K	X	S4

Sample ID	Location	Region	GPS coordinates		GenBank Accession Number	mtDNA COII Haplotype	Genotyped Microsatellites	STRUCTURE Cluster
			Lat	Lon				
Fr28	St Martin de Macon	-	-0.11416700	47.01440000	JQ280555	A	X	S4
Fr29	Thouars	-	-0.21416700	46.97583290	JQ280587	A	X	S4
Fr30	Ciron Château de Romefort	-	1.23750000	46.62222200	JQ280558	A	X	S4
Fr31	Poitiers	-	0.33611200	46.58194500	JQ280573	A	X	S4
Fr32	Jazeneuil	-	0.06972190	46.46583300	JQ280588	A	X	S4
Fr33	La Couarde	-	-0.15930010	46.32000000	JQ280589	A	X	S4
Fr34	Niort	-	-0.46055600	46.32583300	JQ280556	A	X	S4
Fr35	Olonnes	-	-1.82749990	46.54944000	JQ280590	A	X	S4
Fr36	Ile de Ré	-	-1.43377990	46.20130000	JQ280591	A	X	S5
Fr37	La Rochelle	-	-1.14996000	46.16003700	JQ280549	A	X	S5
Fr38	Ile d'Aix	-	-1.17249990	46.01280000	JQ280574	C	X	S5
Fr39	Ile d'Oléron Boyardville	-	-1.33166700	45.97972200	JQ280594	A	X	S4
Fr40	Ile d'Oléron Saumonard	-	-1.24052900	45.97316800	JQ280593	C	X	S4
Fr41	Ile d'Oléron Sables-Vigniers	-	-1.37841200	45.96117400	JQ280592	C	X	S4
Fr42	Ile d'Oléron St Trojan	-	-1.24630500	45.85794500	JQ280597	C	X	S4
Fr43	Ile d'Oléron St Trojan	-	-1.23995000	45.84185900	JQ280598	A	X	S4
Fr44	Rochefort	-	-0.95878600	45.94211100	JQ280601	A	X	S4
Fr45	La Coubre	-	-1.22812300	45.77558900	JQ280595	C	X	S4
Fr46	La Tremblade	-	-1.14166700	45.76944400	JQ280542	C	X	S5
Fr47	Saintes	-	-0.63330000	45.74640000	JQ280602	C	X	S4
Fr48	St Georges de Didonne	-	-0.99783800	45.60271600	JQ280545	C	X	S5
Fr49	Mortagne sur Gironde	-	-0.78361100	45.48361100	JQ280546	A	X	S4
Fr50	Vindelle	-	0.12249990	45.72110000	JQ280557	A	X	S5
Fr51	Jarnac	-	-0.17580000	45.68140000	JQ280540	A	X	S4
Fr52	Galgon	-	-0.27138900	44.99222200	JQ280547	C	X	S4
Fr53	St-Loubes	-	-0.42694390	44.91810000	JQ280563	I	X	S4

Sample ID	Location	Region	GPS coordinates		GenBank Accession Number	mtDNA COII Haplotype	Genotyped Microsatellites	STRUCTURE Cluster
			Lat	Lon				
Fr54	Pessac	-	-0.63111100	44.80666700	JQ280565	H	X	S4
Fr55	Talence	-	-0.58400000	44.80000000	JQ280564	A	X	S4
Fr56	St-Médard en Jalles	-	-0.71638900	44.89638900	JQ280544	C	X	S4
Fr57	St-Médard en Jalles	-	-0.71638900	44.89639000	JQ280548	C	X	S4
Fr58	La Teste-de-Buch	-	-1.14571100	44.62000000	JQ280551	C	X	S4
Fr59	Arcachon	-	-1.16918600	44.65278000	JQ280550	C	X	S5
Fr60	St-Michel de Montaigne	-	0.03083310	44.87500000	JQ280560	A	X	S5
Fr61	Castillon-la-Bataille	-	-0.04333300	44.85416700	JQ280543	A	X	S5
Fr62	Bergerac	-	0.48305500	44.85194390	JQ280561	J	X	S5
Fr63	Eymet	-	0.39888900	44.66861100	JQ280553	A	X	S5
Fr64	Montauban	-	1.35583290	44.01805600	JQ280541	E	X	S5
Fr65	Montans	-	1.88611100	43.86722200	JQ280539	B	X	S5
Fr66	Albi	-	2.14638900	43.92888900	JQ280599	B	X	S5
Fr67	St Pierre d'Irube	-	-1.45777800	43.47719990	JQ280562	C	X	S5
Fr68	Anglet	-	-1.51833400	43.48499990	JQ280559	C	X	S5
Fr69	Toulouse	-	1.44396190	43.60448200	JQ280552	G	X	S5
Fr70	Martigues	-	5.04750000	43.40527800	JQ280600	A	X	S5
URU	Montevideo, Uruguay 2	-	-	-	AY808080	A	-	-
USA	Sacramento California	-	-	-	AY808087	H	-	-
USA3	Nebraska 1	-	-	-	JQ280698	A	-	-
USA5	Iowa	-	-	-	JQ280704	A	-	-
USA8	Indiana	-	-	-	JQ280705	A	-	-
USA10	West of Virginia 1	-	-	-	JQ280728	CB	-	-
USA11	West of Virginia 2	-	-	-	JQ280729	W	-	-
USA17	Delaware	-	-	-	JQ280725	BZ	-	-
USA22	North East of Virginia 3	-	-	-	JQ280735	CC	-	-

Sample ID	Location	Region	GPS coordinates		GenBank Accession Number	mtDNA COII Haplotype	Genotyped Microsatellites	STRUCTURE Cluster
			Lat	Lon				
USA28	North Carolina 3	-	-	-	JQ280718	BV	-	-
USA38	Toronto	-	-	-	AF525324	K	-	-
MI56	Mississippi	-	-	-	JQ280623	W	-	-
MI61	Mississippi	-	-	-	JQ280742	A	-	-
FL3	Gainsville Florida 3	-	-	-	JQ280668	AN	-	-
FL19	Wakulla Florida 1	-	-	-	JQ280665	A	-	-
LA2	New Orleans Louisiana 2	-	-	-	JQ280738	H	-	-
LA3	New Orleans Louisiana 3	-	-	-	JQ280739	K	-	-
LA4	New Orleans Louisiana 4	-	-	-	JQ280740	A	-	-
LA5	New Orleans Louisiana 5	-	-	-	JQ280608	E	-	-
LA32	NLCr Louisiana 4	-	-	-	JQ280612	C	-	-

Table S2. Description of *Reticulitermes flavipes* samples collected from in and around Tours: sampling locations and GPS coordinates (latitude/longitude).

Sample ID	Location	GPS coordinates	
		Lat	Lon
**Tours**			
TCT01	Allée Pr G. Louis	0.695303	47.359534
TCT02	Allée J. de la Bruyère	0.694249	47.359721
TCT03	Allée J. de la Bruyère	0.693971	47.359811
TCT04	Allée J. de la Bruyère	0.693946	47.360151
TCT05	Allée J. de la Bruyère	0.693904	47.360420
TCT06	Allée J. de la Bruyère	0.693762	47.360717
TCT07	Allée J. de la Bruyère	0.693902	47.360997
TCT08	Allée J. de la Bruyère	0.693935	47.360907
TCT09	Allée J. de la Bruyère	0.694258	47.360896
TCT10	Allée J. de la Bruyère	0.694274	47.360410
TCT11	Allée Pr G. Louis	0.695283	47.359898
TCT12	Rue Bergeonnerie	0.694774	47.359074
TCT13	Allée J. de la Bruyère	0.693725	47.361527
TCT14	Allée J. de la Bruyère	0.693781	47.361768
TCT15	Allée J. de la Bruyère	0.694171	47.362130
**Joué les Tours**			
TCJT01	Parc de la Rabière	0.671906	47.34668
TCJT02	Parc de la Rabière	0.671869	47.346523
TCJT03	Parc de la Rabière	0.671634	47.346157
TCJT04	Parc de la Rabière	0.671609	47.345956
TCJT05	Parc de la Rabière	0.671402	47.345772
TCJT06	Rue de la rabière	0.666619	47.345647
TCJT07	Rue Verdun	0.665254	47.346108
TCJT08	Rue Verdun	0.666043	47.346282
TCJT09	Rue Verdun	0.665917	47.346431
TCJT10	Rue Verdun	0.665732	47.346712
TCJT11	Rue Verdun	0.665874	47.346844
**La Riche**			
TCLR01	Chemin du Vivier	0.642519	47.378285
TCLR02	Chemin du Vivier	0.641321	47.377959
TCLR03	Rue des Montils	0.641614	47.377159
TCLR04	Chemin du Vivier	0.642656	47.377928
TCLR05	Rue des Montils	0.642832	47.377237
TCLR06	Rue des Montils	0.643455	47.376065
TCLR07	Rue des Montils	0.642518	47.376374

Table S3. Haplotype diversity of the mitochondrial gene COII in *Reticulitermes flavipes* in France

	n	*Nh*	*Hd*	*Nd (Pi)*
**Region**				
Ile de France	63	6	0.561 ± 0.047	0.0032
Centre-Val de Loire	62	4	0.531 ± 0.053	0.0024
**France**	175	12	0.720 ± 0.024	0.0047

Sample size *(n)*, number of haplotypes identified *(Nh)*, haplotype diversity (*Hd*), and nucleotide diversity (*Nd*)

Table S4. Genetic differentiation among the five *Reticulitermes flavipes* clusters in France identified by STRUCTURE using eight microsatellite loci (n = 164 workers). Pairwise population *F*st values were obtained after 1,000 permutations.

	**S2**	**S3**	**S4**	**S5**
**S1**	0.26*	0.49*	0.27*	0.21*
**S2**		0.47*	0.28*	0.25*
**S3**			0.47*	0.48*
**S4**				0.11*

The asterisks indicate significant differences were present at an adjusted significance level ($p<0.005$; original alpha = 0.05).
